# Prevalence, distribution, and antibiotic resistance of bacterial isolates in daycare centers in Ile-Ife, Nigeria

**DOI:** 10.1017/ash.2024.473

**Published:** 2024-12-20

**Authors:** Eunice Damilola Wilkie, Jude Oluwapelumi Alao, Oluwakemi Abike Thonda, Anthonia Olufunke Oluduro

**Affiliations:** 1 Microbiology Department, Adeleke University, Osun State, Nigeria; 2 School of Public Health and Interdisciplinary Studies, Auckland University of Technology, Auckland, New Zealand; 3 Department of Microbiology, Babcock University, Ilishan-Remo, Ogun State, Nigeria; 4 Department of Microbiology, Obafemi Awolowo University, Ile-Ife, Nigeria

## Abstract

This study investigates bacterial pathogen prevalence in daycare centers in Ile-Ife, Nigeria, highlighting significant contamination and high antibiotic resistance rates, particularly among *Staphylococcus aureus*. Findings underscore the need for enhanced infection control measures, sanitation practices, and public health interventions to protect children’s health in resource-limited settings.

## Introduction

Daycare centers, increasingly utilized worldwide for early childhood care, present environments that can facilitate the transmission of infectious diseases among children. In developing countries like Nigeria, poor sanitation, limited access to clean water, and inadequate hygiene practices exacerbate this risk.^1^ Understanding the prevalence of bacterial pathogens in daycare settings is crucial to devising public health interventions that protect children’s health.

Children’s susceptibility to infections stems from developing immune systems, and common pathogens such as *Staphylococcus aureus*, *Escherichia coli*, and *Streptococcus* species can lead to illnesses ranging from mild skin infections to severe respiratory or gastrointestinal diseases.^2^ The spread of antibiotic-resistant bacteria in these settings further complicates treatment options, elevating healthcare costs and public health risks.

This study focuses on bacterial pathogen prevalence in daycare centers in Ile-Ife, a city in southwestern Nigeria. It aims to quantify bacterial isolates in various daycare samples, identify environmental and behavioral factors contributing to contamination, and compare findings with other regions to understand public health implications better.

## Methods

### Ethics and sampling

Approval for the study was obtained from the Health Research Ethics Committee of Obafemi Awolowo University (HREC Number: IPHOAU/12/1337). Samples were collected from 20 daycare centers, both urban and rural, from 2017 to 2019. Collection sites included fomites (toys, diaper changing areas, mats, etc.), children’s hands and nostrils, and daycare workers’ hands. Consent was obtained from parents and daycare workers before sample collection.

### Bacterial isolation and identification

Swabs were transported to a laboratory, where bacterial isolation was conducted using Nutrient agar, EMB agar, MacConkey agar, and Blood agar plates. Biochemical identification tests, such as the catalase, citrate, and oxidase tests, were employed to characterize isolates. In addition, the Microbact^TM^ GNB 24E identification kit was used for gram-negative isolates, providing a detailed metabolic profile.

### Antibiotic susceptibility testing

Antibiotic susceptibility was determined using the Kirby–Bauer disk diffusion method and the automated VITEK 2 system. Resistance patterns were established based on the Clinical and Laboratory Standards Institute (CLSI) criteria,^3^ focusing on commonly used antibiotics like gentamycin, ampicillin, and ciprofloxacin.

### Data analysis

Data analysis included calculating prevalence percentages to understand the distribution of resistance patterns. χ^2^ tests were selected to evaluate associations for categorical variables, as this test is practical for examining relationships in categorical data. For continuous variables, ANOVA was considered to compare group means, which could provide insights into variability across sources. The Shapiro–Wilk test was applied to verify the assumption of normality required for ANOVA. Statistical significance was defined at *P* < 0.05 across all tests.

## Results

### Baseline characteristics and bacterial distribution

A total of 233 samples were analyzed, comprising 76 from children, 33 from workers, and 124 fomites. *Bacillus species* were the most prevalent, constituting 51.55% of isolates, particularly from fomites and nostrils of children aged 13–24 months. The next most common isolates were *S. aureus* and *Corynebacterium xerosis*.

Gender analysis revealed that bacterial isolates were more frequently found on the hands of male children, with *Bacillus sp.* being the most common. *Bacillus* and *Staphylococcus sp.* were the predominant isolates in workers’ samples, indicating cross-contamination risks [7].

### Antibiotic resistance and ESBL production

**Figure 1.**
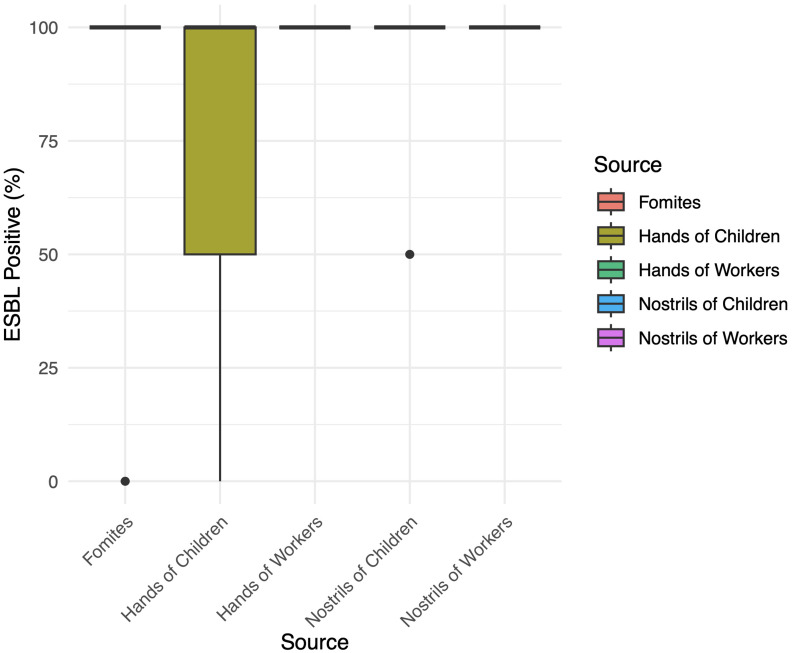
ESBL positive rates by source.

High antibiotic resistance was observed in *S. aureus* isolates, with resistance rates reaching 85.7% for ampicillin and Augmentin, while gentamycin and ciprofloxacin remained effective. The presence of extended-spectrum beta-lactamase (ESBL)-producing strains was notable, with ESBL positivity at 80% in fomites and 100% in workers’ samples. Multiple antibiotic resistance (MAR) indexing revealed significant resistance across multiple antibiotics, particularly in fomites and worker samples. ANOVA analysis indicated no significant association (*P* >.05) between ESBL production and source, with a 95% family-wise confidence level, and results from the post hoc Tukey’s HSD analysis are presented in Figure 1. Analysis of continuous variables for normality revealed a significant departure from normal distribution (W = 0.40235, *P* = 2.997e–09), leading to using the Kruskal–Wallis test for nonparametric analysis. The Kruskal–Wallis rank sum test showed no significant differences in ESBL positivity across sources (2.741, df = 4, *P* value = .6021), indicating a consistent distribution of ESBL production across the different sources examined.

## Discussion

This study highlights the critical role of daycare centers as potential reservoirs and transmission hubs for infectious diseases, particularly in resource-limited settings. A high prevalence of *Bacillus sp.* was observed, reflecting the resilience of these spore-forming bacteria in environments where hygiene standards may fluctuate. This aligns with findings in similar studies conducted in Nigerian daycare settings,^4,5^ which also report significant prevalence rates of *S. aureus* and *Bacillus sp.*, underscoring the need for stricter infection control measures in such environments.

The high prevalence of pathogenic organisms, such as *S. aureus*, with resistance rates of 85.7% to antibiotics like ampicillin and Augmentin, poses significant public health risks and is consistent with other studies from Nigeria and other developing regions.^6,7^ These pathogens are associated with severe skin infections, respiratory illnesses, and gastrointestinal diseases. ESBL-producing strains, with positivity rates of 80% in fomites and 100% in workers’ samples, further complicate treatment efforts. The combination of high antibiotic resistance and the emergence of ESBL-positive strains indicates a pressing challenge for healthcare providers, necessitating rigorous infection control measures and targeted public health interventions to protect vulnerable populations, particularly young children whose immune systems are still developing.

In contrast, while still concerning, the lower prevalence of organisms with low pathogenicity, such as *Bacillus* and *Corynebacterium*, implies a different risk profile. For instance, *Bacillus sp*., which accounted for 51.55% of the isolates, are often resilient and can persist in environments with variable hygiene. Their presence, particularly in fomites and children’s hands, highlights the need for improved sanitation and hygiene practices in daycare settings. Although low-pathogenicity organisms generally pose a lower risk of severe illness, they can contribute to overall bacterial load. They may facilitate the spread of antibiotic resistance when they harbor resistance genes. Moreover, the distinction between these bacterial groups is crucial. At the same time, pathogenic strains necessitate immediate intervention, and low-pathogenicity organisms may require a focus on education and hygiene improvements to prevent their proliferation.

Contrasting these findings with studies from high-income countries reveals significant disparities, with markedly lower rates of pathogenic bacterial prevalence and antibiotic resistance likely attributable to stricter hygiene, sanitation protocols, and more regulated antibiotic use.^8,9^ This contrast underscores the pressing need to address antibiotic resistance in resource-limited settings, where treatment options for resistant infections remain limited. Improvements in hygiene practices, regulated antibiotic use, and routine antimicrobial surveillance in daycare facilities could mitigate the spread of these resistant strains.

This study’s scope was limited by the absence of molecular genetic testing and other microbiological assays that could have provided more comprehensive profiling of resistance genes. Additionally, broader surveillance across multiple centers would provide a more thorough understanding of the prevalence and resistance patterns in the region, allowing for better-informed public health strategies. These limitations should be considered when generalizing the findings, and future studies are encouraged to integrate molecular resistance profiling and a more extensive sample base to enhance the robustness of results.

## Conclusion

The significant bacterial contamination and high antibiotic resistance rates in Ile-Ife daycare centers highlight an urgent need for infection control measures, stricter sanitation practices, and public health policies. Educational programs for daycare staff and routine monitoring of hygiene practices could mitigate bacterial spread. Our findings call for a multifaceted approach to protect children’s health, incorporating regular screenings and controlled antibiotic use.
